# Newborn Care Practices among Mother-Infant Dyads in Urban Uganda

**DOI:** 10.1155/2015/815938

**Published:** 2015-12-02

**Authors:** Violet Okaba Kayom, Abel Kakuru, Sarah Kiguli

**Affiliations:** ^1^Department of Paediatrics and Child Health, Makerere University College of Health Sciences, Kampala, Uganda; ^2^Infectious Diseases Research Collaboration, Kampala, Uganda

## Abstract

*Background*. Most information on newborn care practices in Uganda is from rural communities which may not be generalized to urban settings.* Methods*. A community based cross-sectional descriptive study was conducted in the capital city of Uganda from February to May 2012. Quantitative and qualitative data on the newborn care practices of eligible mothers were collected.* Results*. Over 99% of the mothers attended antenatal care at least once and the majority delivered in a health facility. Over 50% of the mothers applied various substances to the cord of their babies to quicken the healing. Although most of the mothers did not bathe their babies within the first 24 hours of birth, the majority had no knowledge of skin to skin care as a thermoprotective method. The practice of bathing babies in herbal medicine was common (65%). Most of the mothers breastfed exclusively (93.2%) but only 60.7% initiated breastfeeding within the first hour of life, while a significant number (29%) used prelacteal feeds.* Conclusion*. The inadequate newborn care practices in this urban community point to the need to intensify the promotion of universal coverage of the newborn care practices irrespective of rural or urban communities and irrespective of health care seeking indicators.

## 1. Introduction

Neonatal morbidity and mortality have remained high in most developing countries and are affecting their attainment of Millennium Development Goal (MDG) 4. Neonatal sepsis is one of the major causes of these deaths, most of which are preventable [1, 2]. Studies in developing countries have shown strong evidence that delivery and newborn care practices impact on neonatal sepsis [3–7]. In an effort to combat these alarming rates, World Health Organization (WHO) has put forth a set of evidence based neonatal health interventions called the essential newborn care (ENC) practices. They include clean delivery, cord care, skin and eye care, early and exclusive breastfeeding, thermal protection, and immunisation [8]. These interventions have been widely emphasized in policy and rolled out in developing countries.

Uganda is grappling with a high neonatal mortality rate of 27 per 1000 live births with neonatal sepsis being the major contributor [9]. Unhygienic delivery and poor newborn care practices coupled with poor accessibility to health facilities in rural areas have been cited as key risk factors [9, 10]. Although urban communities in Uganda have better accessibility to health facilities and higher rates of antenatal care (ANC) attendance (98%) and report more deliveries under skilled supervision [9], health facilities in urban settings continue to receive large numbers of neonates presenting with symptoms of neonatal sepsis. A survey done in Mulago National Referral Hospital in Uganda in 2012 showed that neonatal sepsis was the most common cause of admission to the neonatal ward (unpublished data). Most of these neonates were born in health facilities and discharged home, only to return to hospital with symptoms of neonatal sepsis. This finding suggests that there are unexplained risk factors not only in the health facilities but also in the communities to which these neonates are discharged.

Most studies on newborn care practices have been conducted in rural settings and these studies have reported disturbing rates of poor delivery and newborn care practices [11, 12]. There is a gap in information on newborn care practices in urban settings which are usually cross-cultural and better served with health care, yet reporting high rates of admission with neonatal sepsis. This study was therefore conducted in the capital city of Uganda and it sought to describe the newborn care practices among mothers in that community.

## 2. Methodology

### 2.1. Study Design

This was a community based cross-sectional descriptive study.

### 2.2. Study Site

The study was conducted in Kawempe division, one of the divisions of Kampala city which is the capital city of Uganda. Kawempe division has an area of 32.45 square kilometers and is administratively made up of 22 parishes and 119 zones. It is a densely populated area and according to the 2002 Population and Housing Census its population estimate was 268,659 of which 52% were female. The total number of households was 67,132. The division has several areas with uncontrolled developments and slum conditions. It is served by the National Referral Hospital, 2 government health centres, a private-not-for-profit hospital, and many privately owned clinics which provide curative services to the community [13].

### 2.3. Study Population

The study included all mothers of neonates residing within Kawempe division during the study period who met the selection criteria.

### 2.4. Study Duration

The study was conducted from February to May 2012 (4 months).

### 2.5. Inclusion Criteria

The inclusion criteria included all mothers of neonates living within Kawempe division during the study period who accepted to give informed consent to participate in the study.

### 2.6. Exclusion Criteria

The exclusion criteria included mothers of babies with gross congenital malformation.

### 2.7. Samples Size

The sample size was calculated using the modified Kish Leslie formula:

(1) $$\begin{array}{c} N=\frac{\left(Z^{2*}P\left(1-P\right)*Deff\right)}{D^{2}}, \end{array}$$

where *Z* is standard normal value corresponding to 95% confidence interval (1.96), *D* is absolute error between the estimated and true value which equals 0.05 (5%), *P* is estimated prevalence of poor newborn care practices which is 17%, and Deff represents design effect taken to be 1.5.

Hence, *N* = 325 neonates.

### 2.8. Number of Clusters

The number of clusters, *C*, that was studied was calculated from the following formula [14]:

(2) $$\begin{array}{c} C=\frac{P\left(1-P\right)D}{S^{2}b}, \end{array}$$

where *P* is prevalence, *D* is design effect (1.5), *S* is standard error given by confidence interval/*Z* alpha (0.05/1.96 = 0.0255), and *b* is number of responses per cluster set at 10 for convenience.

The estimated number of clusters was 33. To allow for nonresponse a total of 34 clusters were studied. Thus the sample size calculated was* 335* mothers with neonates.

### 2.9. Data Collection

Probability proportional to size was used to sample 34 villages out of 119 villages within Kawempe division in Kampala. The research team held meetings with chairpersons of the Local Council 1 and a member of the Village Health Team (VHT) of each sampled village to discuss the study. The VHT member guided the study team to the households in each village and households with neonates were identified. Informed consent was obtained from the eligible mothers of the neonates and a total of 10 households with neonates were consecutively enrolled from each village. A pretested questionnaire was used to record information on the characteristics of study participants and newborn care practices of the mothers. The gestational age and birth weight of the neonates were estimated by self-report. The newborn practices assessed included cord care (number of times the umbilical stump was cleaned in a day and what was applied on the umbilical stump), skin care (number of times the baby's skin was cleaned in a day, washing of hands prior to handling the baby, and use of herbal medicine to bath baby), early and exclusive breastfeeding, thermal protection, and immunisation.

The newborn care practices were further explored in a focus group discussion (FGD) which was conducted on completion of enrolment of the 338 study participants. Nine participants were included in the FGD. These were mothers of neonates who were identified from 2 zones within the study area by the VHTs with guidelines from the study team. Their ages ranged between 21 and 34 years. The FGD was moderated by the principal investigator assisted by a note-taker and the interviews were conducted in the local language of the majority of participants (*Luganda*) and English. The responses were translated into English by the note-taker and the principal investigator and the information was cross-checked to confirm the meanings.

### 2.10. Data Management and Analysis

All data was entered, cleaned, edited, coded, and double entered into ACCESS data base 2007. It was stored in both manual and electronic forms. Quantitative data was exported to STATA version 10 for analysis. Categorical variables were summarised into percentages and proportions.

Qualitative data from the FGD was stored in manual form. The information was compared and meaningful units were coded. These were broadly categorised into themes. Relevant quotes were extracted and represented.

### 2.11. Ethical Considerations

Written informed consent was obtained from the mothers of infants who were eligible.

Institutional approval was sought from Makerere University School of Medicine Research and Ethics Committee and the Uganda National Council for Science and Technology.

### 2.12. Study Profile

During the study period, a total of 358 mother-infant pairs were screened for the study. Fifteen did not consent to participate in the study, while 5 babies were found to be over 28 days. Therefore 338 mother-infant pairs were enrolled in the study.

## 3. Results

### 3.1. Description of the Study Participants

Twenty-six percent of the mothers were prime gravida. Their mean age was 25.4 years (sd 5.4).

Most of the mothers were married and received financial support from their husbands. Over 99% of the mothers attended ANC at least once and the majority delivered in a health facility (Table 1).

Most of the neonates were born at term and the mean gestational age was 39.7 weeks (sd 2.3) with mean birth weight of 3.4 kg (sd 0.7). Thirty-two (9.5%) of the neonates had low birth weight. The neonates were equally distributed by gender with males comprising 156 (46.2%) and the mean age at enrolment was 14.8 days (sd 8.5).

### 3.2. Newborn Care Practices of Mother-Infant Dyads

The newborn care practices assessed in this study included cord care, thermal protection, skin care, early and exclusive breastfeeding, and immunisation.

#### 3.2.1. Cord Care

More mothers reported that they had received teachings on cord care (69.5%) although few (45%) had received the teachings from health personnel. Some mothers reportedly received teachings from their attendants, neighbours, and fellow mothers. Majority of the mothers cleaned the cord at least twice a day (89.6%) and washed their hands prior to cleaning the cord (84.9%). About 58% of the mothers applied various substances on the cord of their babies (Table 2). These substances included salty water, powder, Vaseline, spirit, normal saline, ripe banana (*gonja*), sap, soot, ash, saliva, and herbs.

Mothers who delivered in private clinics were 1.2 times more likely to apply substances to the cord compared to those who delivered in hospital. Incidentally there was no significant difference in the practice of application of substances to cord among mothers who delivered at home and those who delivered in hospital. The level of education, parity, and the number of ANC attendances did not result in any significant difference in the practice of application of substances to the cord (Table 3).

From the qualitative data majority of the mothers reported that it was important to apply substances to the cord to quicken the healing. The commonly used substance was warm salty water.

*FGD member said, “I clean the baby's cord with clean cotton wool dipped in warm salty water which I keep in a bottle.”*

*FGD member said, “Spirit helps the cord dry faster.”*

#### 3.2.2. Thermal Protection

About sixty percent of the mothers did not bathe their babies within the first 24 hours of birth. However, most of the mothers had no knowledge of skin to skin care or kangaroo mother care (KMC) as a way of keeping babies warm (77%). Only 28% of the mothers with low birth weight or preterm babies practiced KMC (Table 2).

Mothers who delivered in health centres and private clinics were twice more likely to bathe their babies within 24 hours of birth compared to those who delivered in hospital. Incidentally those who delivered at home were 1.5 times less likely to bathe their babies within 24 hours of birth compared to those who delivered in health centres or private clinics (Table 3).

In the qualitative data, all the mothers acknowledged the importance of keeping babies warm in the first 24 hours. However, some of the reasons given for thermal protection were not the scientifically valid reasons, while other mothers did not know why they delayed bathing their babies. Examples of misconceptions and lack of knowledge about reason for practices are reflected in the quotes below.

*FGD member said, “You keep the baby warm because you do not want cold air to enter the baby's lungs since it can cause pneumonia.” *

*FGD member said, “I bathe my baby 2 days after birth because the baby collapses in water if you bathe early.”*

*FGD member said, “I do not know why I have to wait for 2 days to bathe my baby.”*

#### 3.2.3. Skin Care

Majority of mothers washed their hands prior to handling their babies (83.4%) and cleaned their babies at least twice in a day (90.2%). The practice of bathing babies in herbal medicine was common in majority of the respondents, that is, 205 (60.7%). In the qualitative study, there were conflicting views with regard to bathing babies in herbal medication which was called “*ekyogero*.” This was said to be a mixture of various herbs including leaves, roots, and tree barks. The herbal medications were used to prevent and treat skin rashes (locally called “*nnoga*”), treat abdominal colic, and give the baby good luck. Examples of practices and beliefs about the use of herbal medicines to bathe the baby as reported in the FGD are shown by the quotes below.

*FGD member said, “Before you bathe the baby in ekyogero you drop some of the fluid in the baby's mouth. This prevents the baby from suffering from stomache [sic] pain.”*

*FGD member said, “Bathing the baby in ekyogero makes the baby get good luck. Anyway other children are not bathed in it but they get their blessings from God.”*

Other members did not agree with the practice of bathing babies in herbal medicine mainly due to cultural differences.

*FGD member said, “For us the Bakiga we do not bathe our babies in ekyogero, we just bathe the baby with soap and apply powder and vaseline. But look we are among the luckiest people….”*

#### 3.2.4. Breastfeeding

Most of the mothers breastfed their babies exclusively (93.2%), while only 60.7% initiated breastfeeding within the first hour of life (Table 2). Over twenty-nine percent (29.6%) of the mothers used prelacteal feeds which included glucose solution, cow's milk, water, and tea.

Mothers who delivered in hospitals or health centres were 1.2 times more likely to initiate breastfeeding within the first hour of life compared to those who delivered at home or in private clinics. Level of education, parity, and number of ANC attendances were not found to significantly affect time to initiation of breastfeeding (Table 3).

In the FGD there were varying views about the time to initiate breastfeeding following delivery. Some mothers said it was important to put the baby to breastfeed immediately after birth, while others thought there were no strict timelines. It was also reported that health workers take the babies away for long and this delays initiation of breastfeeding. Most mothers thought it was okay to give prelacteal feeds although a few strongly objected. Examples of breastfeeding practices and misconceptions are reflected in the quotes below.

*FGD member said, “You should put the baby immediately on the breast even if there is no milk.” *

*FGD member said, “The health workers sometime take the baby away from you, so it is okay to wait.” *

*FGD member said, “You give the baby warm water with glucose because you may have no breast milk….” (Most mothers agreed to this.)*

#### 3.2.5. Immunisation

Seventy-six percent of the babies had received their Bacille Calmette-Guérin (BCG) and Oral Polio Vaccine (OPV) at the time of contact with the study team.

## 4. Discussion

This study was conducted to determine the newborn care practices among mother-infant dyads in an urban community in Uganda. Although some components of the evidence based newborn care practices in this study were appropriate, the overall practices were deemed inadequate.

### 4.1. Cord Care

In our study, majority of the mothers cleaned the cord frequently and washed their hands prior to cleaning the cord. However, the practice of applying substances on the cord was highly prevalent. It was quite surprising to find that the practice of application of substances to the cord was equally prevalent among mothers who delivered in health facility and those who delivered at home. This suggests that the teachings on appropriate cord care at the health facilities are not adequate or the uptake is poor possibly due to cultural beliefs. From the qualitative data it was clear that most mothers believe that various substances should be applied to the cord to hasten healing of the stump. This finding is similar to that reported in rural Eastern Uganda in which about half of the mothers applied various substances to the cord [11]. However, a study in urban Nepal reported much better cord care with 86% of the mothers who had delivered in health facilities not applying any substance to the cord and rates of 63% of those who had delivered at home [15]. The difference in the findings may be due to cultural variations between the urban community in our study and that in Nepal. WHO currently recommends that the cord be cleaned with daily chlorhexidine for babies born at home in high mortality settings. For babies born in health facilities or at home in low mortality settings it may be considered to replace application of harmful traditional substances [16]. Our study indicates that there are a knowledge gap and misconceptions on care of the cord in this urban community and the evidence based recommendations have not been taken up. It is very surprising that although over 90% of the mothers in our study attended ANC and the majority delivered in a health facility, only about 45% received teachings on appropriate cord care from health personnel. There is no doubt that the mothers are not knowledgeable on the various aspects of appropriate cord care since they do not have a clear expert source of information. The health facilities should therefore step up the health education on appropriate cord care during ANC and in the period after delivery. In addition, WHO recommendation for chlorhexidine cleansing should be extended fully to all babies in settings with high mortality and high infection regardless of place of birth.

### 4.2. Thermal Protection

Most of the mothers in this community did not have any knowledge of skin to skin care or KMC, and even for mothers with low birth weight or preterm babies most did not practice KMC. KMC has been shown to reduce neonatal mortality among low birth weight and preterm babies and reduce severe morbidity [3]. This practice should be widely scaled up especially in high mortality, low resource settings.

Some components of good thermal protection were practiced by majority of the participants. These included delayed bathing for more than 24 hours and covering babies in warm clothes immediately after birth. Although mothers who delivered in hospitals were less likely to bathe their babies within 24 hours of birth compared to those who delivered in health centres or private clinics, mothers who delivered at home were equally less likely to bathe their babies within 24 hours of birth. This finding shows that place of delivery does not necessarily result in good thermal practice and thus the promising finding of delayed bathing in majority of the mothers may simply be due to cultural practice and may not necessarily be a result of health facility promotion of the evidence based practices. This therefore questions the adequacy of teachings on thermal protection. WHO recommends that babies be bathed after 24 hours of birth and not earlier to avoid hypothermia [8]. A study in rural eastern Uganda reported that only 42% of the neonates received adequate thermal protection. In that community early bathing was practiced because of the cultural belief that babies are born dirty [11]. Thermal protection in various communities in Africa seems to vary mainly due to cultural differences. A large cross-sectional study in Tanzania reported that two-thirds of the mothers bathed their babies within 6 hours of delivery and 10% reported that their babies were dipped in cold water immediately after birth [17]. The health facility delivery rate in that study was 40% suggesting poor health seeking behaviour which could also explain the poor thermal protection practice. The subjects in our study had diverse cultural origins and this possibly could have had a dilutional effect on the practice, thus exposing the participants in our study to better thermal protection practice.

Although most of the mothers acknowledged the importance of keeping the baby warm, the qualitative data shows that the majority did not know the scientifically valid reasons why these practices are encouraged. Some mothers perform practices which are important in thermal protection but they do not know that these practices are actually meant to keep the baby warm. An example is delayed bathing of the baby after birth. If mothers do not know why they are being told to do certain things, they may not own the practices and this may affect compliance with the evidence based practices.

### 4.3. Skin Care

Majority of the mothers in our study cleaned their babies two or more times a day. However, the practice of bathing babies in herbal medications (*ekyogero*) was common. The herbal medication is a concoction of various roots, leaves, and stems of plants and is used to bathe the baby, applied to the cord, and given to the baby to drink. The use of this herbal medication has been reported in other communities in Uganda. The health impact of such a practice is not well investigated, yet it is strongly embedded in the culture of this urban community. Further studies on the safety and health effect of use of the herbs on the newborn are therefore warranted.

Hand washing was practiced by a significant number of mothers in our study. Maternal and birth attendant hand washing has been shown to reduce infection in neonates [5] and so its promotion should be intensified in the community.

### 4.4. Early Initiation and Exclusive Breastfeeding

Majority of the mothers in our study practiced exclusive breastfeeding suggesting that some components of the ENC practices have been embraced well in this community. This study reports better exclusive breastfeeding rates compared to that reported in Southern Tanzania in which less than half breastfed exclusively in the 3 days after delivery [17]. The difference in these results may be due to the better health facility delivery in our study.

Our study showed that just over half of the mothers initiated breastfeeding within the first hour of life and about a third gave prelacteal feeds. The finding that mothers who delivered in hospitals and health centres were more likely to initiate breastfeeding within the first hour of life compared to those who delivered in private clinics or at home suggests that there is inadequate promotion of the ENC practices in private facilities and the community. A study in Ghana showed that early initiation of breastfeeding could avert 22% of neonatal deaths [4]. WHO discourages the use of prelacteal feeds unless medically indicated because of its association with insufficient milk production, infection transmission, and lactation failure [18]. Our findings show that despite the good exclusive breastfeeding rates some key components of breastfeeding are still lacking and there is a gap in the private health facilities. The promotion of breastfeeding should therefore focus not only on exclusive breastfeeding but also on early initiation of breastfeeding and discourage the use of prelacteal feeds in the community and public and private health facilities.

### 4.5. Immunisation

In our study, 76% of the neonates had received BCG and OPV at the time of contact with the research team. According to the Uganda National Expanded Program for Immunisation (UNEPI) all infants should receive BCG and OPV at birth or within 2 weeks of delivery. According to the Uganda Demographic Health Survey (UDHS) 2011, 96% of neonates in urban settings received BCG as compared to 93% in rural settings [9]. The immunisation rate in our study is therefore lower than that reported in the UDHS 2011. This indicates that a significant number of babies born in health facilities are being discharged without receiving their BCG and OPV vaccinations. This finding suggests that the health facilities do not have constant supply of vaccines or there is inadequate promotion of the vaccination. The Ministry of Health of Uganda (MoH) and health partners should therefore ensure that every public health facility offers immunisation services daily. Community outreach should be supported and promoted to mop up the remnants of infants who missed the vaccinations in health facilities. The private health facilities should also be supported to provide immunisation services.

### 4.6. Overall Newborn Care Practices

This study was conducted in an urban community which is well served with health facilities. The ANC attendance of the mothers was very high and the rate of health facility delivery and skilled birth attendance was almost twice the national rate and much higher than that reported in the studies in rural settings [9, 11, 12]. It is therefore quite surprising that most of the newborn care practices were not any better than those reported in the rural settings. It is clear that the good health seeking indicators of this community have not really translated into gains in the newborn care practices. The MoH and partners should consider reviewing the strategies currently being used to disseminate the newborn care practices in health facilities and communities. This should include review of the content, mode, and intensity of health education on newborn care practices by health workers during ANC, after delivery, and in postnatal follow-up and involvement of VHTs in the newborn care health education. In addition, the health effects of the cultural practice of bathing babies in the herbal concoction and dropping the fluid in the babies' mouths in this community should be investigated.

### 4.7. Study Limitations

The gestational age and birth weight of the neonates were estimated by self-report from the mothers and this may not be accurate.

## 5. Conclusion

Although some components of the evidence based newborn care practices in this urban community were appropriate, the overall practices were deemed inadequate. The strategies which are currently being used to disseminate the newborn care practices are not meeting the stated objectives. There is therefore pressing need to review and intensify the promotion of universal coverage of the newborn care practices irrespective of rural or urban communities, place of delivery, and the health care seeking indicators being reported.

## Figures and Tables

**Table 1:** Characteristics of 338 mothers in Kampala District, Uganda, 2012.

Variable	Frequency (*n*)	Percentage (%)
Attended antenatal care		
Yes	336	99.4
No	2	0.6
Number of ANC attendances		
<4 times	172	51
≥4 times	166	49
Marital status		
Never married	40	11.8
Divorced	11	3.3
Married	287	84.9
Parity		
Prime	88	26.0
2-3	164	48.5
≥4	86	25.5
Maternal education		
None	14	4.2
Primary	124	36.7
Secondary	160	47.3
Tertiary	40	11.8
Received paternal financial support		
Yes	306	90.8
No	32	9.5
Place of delivery		
Hospital	209	61.8
Health centre	70	20.7
Clinic/maternity	38	11.3
Home	21	6.2
Duration of rupture of membranes		
Less than 18 hours	304	89.9
More than 18 hours	34	10.1

**Table 2:** The newborn care practices of 338 mother-infant dyads in Kampala District, Uganda, 2012.

Variable	Frequency (*n*)	Percentage (%)
Applied substance on the cord		
No	141	41.7
Yes	197	58.3
Number of times the cord is cleaned per day		
None	22	6.5
Once	13	3.8
≥2 times	303	89.6
Washing of hands before cleaning the cord		
No	51	15.1
Yes	287	84.9
Taught cord care		
No	103	30.5
Yes	235	69.5
Bathed baby within 24 hours		
No	201	59.5
Yes	137	40.5
Knew skin to skin/kangaroo mother care		
No	261	77.2
Yes	77	22.8
Washing of hands prior to handling the baby		
No	56	16.6
Yes	280	83.4
Number of times the baby is cleaned in a day		
Less than twice	33	9.8
Two or more times	305	90.2
Bathe baby with herbal medicine		
No	113	34.4
Yes	205	60.7
Exclusive breastfeeding		
No	23	6.8
Yes	315	93.2
Initiation of breastfeeding		
Less than 1 hour	205	60.7
More than 1 hour	133	39.3
Use of prelacteal feeds		
No	238	70.4
Yes	100	29.6
Immunisation		
No	79	23.4
Yes	259	76.6

**Table 3:** Levels of selected newborn care practices in selected demographic, antenatal, and delivery characteristics of mother-infant dyads in Kampala district, Uganda, 2012.

	Applied substance to cord		Early initiation of breastfeeding		Bathed baby within 24 hours of birth	
	Yes	No	Yes	No	Yes	No
Parity						
Para 1	52 (60%)	35	41 (48%)	44	32 (42%)	44
Para >1	143 (56%)	114	162 (67%)	79	107 (43%)	141
Education						
None/primary	75 (54%)	63	84 (62%)	51	56 (41%)	81
Secondary/tertiary	124 (62%)	76	125 (66%)	64	83 (42%)	115
Place of delivery						
Hospital	122 (55%)	101	133 (63%)	78	67 (32%)	143
Health centre	37 (54%)	32	46 (64%)	26	55 (67%)	27
Private clinic	24 (65%)	13	21 (58%)	15	22 (61%)	14
Home	11 (58%)	8	10 (55%)	8	9 (45%)	11
ANC attendance						
Less than 4 times	97 (56%)	77	107 (61%)	67	73 (42%)	101
4 or more times	99 (61%)	64	100 (62%)	62	67 (41%)	97

## Abbreviations

ANC: Antenatal care
BCG: Bacille Calmette-Guérin
ENC: Essential newborn care practices
FGD: Focus group discussion
KMC: Kangaroo mother care
MDG: Millennium Development Goal
MoH: Ministry of Health of Uganda
OPV: Oral Polio Vaccine
UDHS: Uganda Demographic Health Survey
UNEPI: Uganda National Expanded Program for Immunisation
VHT: Village Health Team
WHO: World Health Organization.
